# Some Diseases of the Newborn Baby
*An address to Torquay and District Medical Society on 10th February, 1949.


**Published:** 1949-07

**Authors:** A. A. Moncrieff

**Affiliations:** Nuffield Professor of Child Health, University of London


### SOME DISEASES OF THE NEWBORN BABY.*

BY

A. A. Moncrieff, M.D., F.R.C.P.

*Nuffield Professor of Child Health, University of London.*

---

Of 800,000 babies born each year about 17,000 die during the first four weeks of life, and of these deaths roughly half are said to be due to prematurity. Let us assume that few babies actually die from immaturity alone, and ignore this aspect of the subject and concentrate on the other main causes of death as classified by the Registrar-General.

*Congenital Malformations.* At one time it was believed that these were largely due to gene mutation, but it is now appreciated that there are equally important causes amongst the commoner of which are the position of a foetus *in utero*, maternal infection (virus diseases of the pregnant mother), specific dietetic deficiencies in the mother and also the possibility of damage by X-radiation to the ovarian follicles.

*Asphyxia.* The number of newborn children dying from asphyxia is higher than the number of children under fifteen who are killed on the roads. Asphyxia may be due to respiratory failure or actual suffocation. Adrian in his work on action currents has shown that preparation for respiration takes place before birth. Barcroft has shown that the necessary stimulus to commence respiration after birth is small. There are two centres concerned ; the gasping centre and the " $CO_2$ controlled " centre. The first gasp is initiated by the gasping centre and is induced by diminution in the oxygen supply when the cord is cut or the placenta separated. Once the first gasp is taken the " $CO_2$ controlled " centre carries on. The effect of sedative drugs on the respiratory centre must, therefore, be borne in mind. Many babies that die of asphyxia have, in fact, been drowned by premature inhalation of amniotic fluid or mucus and this should always be borne in mind when prescribing drugs for the mother.

*Treatment.* These cases can be largely overcome by having a proper plan for dealing with the baby soon after birth.

As soon as the baby is born, care should be taken that the upper respiratory passages, including the naso-pharynx, are absolutely clear ; this may be accomplished both by swabbing and suction.

* An address to Torquay and District Medical Society on 10th February, 1949.

The baby's laryngeal fissure is so tiny that the space between the cords may be occluded by even a very minute piece of mucus.

Clamp the cord at once to produce physiological stimulus for the gasp centre. It is probable that the last few drops of blood in the cord are of no particular value to the child anyway.

Keep the baby's head low for the first few hours after delivery to prevent aspiration of mucus.

Artificial respiration does no good unless, possibly, given by a rocking machine. The ordinary methods produce trauma only.

*Birth Trauma.* Injuries to the long bones, though regrettable, do not result in death, which is generally produced by cerebral injuries—often following instrumentation. Intracranial hæmorrhage is almost invariably fatal except in a few cases where it is sub-dural. However, after instrumentation there is occasionally œdema and congestion. In such cases the baby is concussed and the symptoms take two to three days to develop, whereas in cerebral injury the symptoms develop at once. Common symptoms are that the child becomes comatose or may show irritability. The fontanelle becomes tense and there may be neck or leg stiffness accompanied by vomiting, respiratory difficulties and reluctance to feed. The treatment for these cases is to keep the child in a dark room and flat. Oxygen should be given, as the danger to the baby is anoxæmia. Good nursing is most essential and the normal feeding should be kept going. Sedatives should be given—chloral is the best. To reduce intracranial pressure, hypertonic saline (2 oz. of 10 per cent. solution) should be run into the rectum two or three times and allowed to stay there until rejected. This line of treatment will prevent death in most borderline cases ; the ultimate effect, however, cannot be foreseen and though many of the cases may become quite normal, some later develop spasticity.

*Infection.* The number of babies dying from infection is probably underestimated ; the picture of infection in infancy is not typical. Two points in particular are not appreciated ; the first is that the very mildest sign of sepsis in the infant indicates danger ; and second, the normal temperature in infants is probably between 96 deg. and 97 deg. and not 98 deg. as in the adult, so that a child showing an apparently normal temperature may have a hyperthermia of 1.5 deg. to 2 deg.

The question now arises of transferred immunity from mother to child. It is possible that placental transference may give some passive immunity to the child against diseases to which the mother is immune, but it is probable that this immunity rapidly passes off. It is uncertain whether any antibodies are transferred via the colostrum. It is certain that many infections to which the mother is not immune may affect the child from birth and this applies in particular to the common cold and pyogenic infections. The common cold virus is probably a common infection in the newborn child but its symptoms are not easily perceived : any baby with diarrhœa and vomiting, tendency to blueness and reluctance to feed is probably infected. If those who are in attendance on the child are examined it is almost certain to be found that one of them has a cold. In such cases if the infant dies it is usually from nasal obstruction, because it is not the habit of babies to breathe through the mouth. Such cases should not arise and it is advisable that all in attendance on the child should wear masks.

So far as the pyogenic infections are concerned, the primary infection may be negligible and may present the minor degree of paronychia, ophthalmia, nasal catarrh or infection of the cord stump. Following such minor degrees there is the general picture of sepsis neonatorum. The baby stops feeding, vomits, ceases to gain weight (all alimentary symptoms). It becomes anæmic and grey ; or there may also be jaundice and œdema of the legs. A short and usually fatal stage is that of pyæmia with pemphigus, boils and bronchopneumonia. Take primary infections seriously and treat at once with penicillin by the mouth. When the stage of sepsis neonatorum develops, penicillin is not only given by the mouth but 4,000 units per pound of body weight per day should be given intramuscularly and in addition sulpha-methazine for five days. It is important to keep the infant on the same foods as it has been having hitherto. The symptoms are those of gastro-intestinal disturbance, so the feeding comes under suspicion and is changed : it is better to avoid this, as the child has enough to cope with as it is, without the added upset of dealing with an unaccustomed diet. Strict watch should be kept on the blood-count and transfusion undertaken if necessary.

